# 182. Clinical Presentation of Patients with *Staphylococcus lugdunensis* Positive Blood Cultures After the Implementation of Rapid Molecular Blood Culture Diagnostics

**DOI:** 10.1093/ofid/ofab466.384

**Published:** 2021-12-04

**Authors:** Kristin Constance, Alauna Hunt, Sam Karimaghaei, Nigo Masayuki

**Affiliations:** 1 University of Texas Medical Center at Houston, Houston, Texas; 2 University of Texas Health Science Center Houston, Humble, Texas; 3 McGovern Medical School, The University of Texas Health Science Center at Houston, Spring, Texas; 4 University of Texas in Houston, Houston, TX

## Abstract

**Background:**

Since the implementation of improved laboratory techniques, coagulase negative *Staphylococcus* (CoNS) have been routinely speciated to screen for *S. lugdunensis (SL),* which has led to increased identification. The objective of this study is to describe the characteristics of patients with *SL* positive blood cultures after the introduction of Verigene® Gram-Positive Blood Culture Nucleic Acid Test (BC-GP) in two large medical systems.

**Methods:**

Retrospective review of all blood culture isolates positive for *SL* from Memorial Hermann Hospital System (14 hospitals) and HarrisHealth System (two acute care hospitals) since implementation of BC-GP.

**Results:**

Between 2017 – 2021, 157 patients had *SL* positive blood cultures. 18 were eliminated as cultures were positive for bacteria other than CoNS, and 7 eliminated as patients were discharged prior to culture results. Of the remaining 132 patients, 39 (29.5%) were labelled contaminants by the treating physician and 93 were considered true bacteremia. Patients with hardware/implanted materials were more likely considered to have true bacteremia, while patients with other CoNS species in blood cultures were more likely considered contaminants. Only one death was attributed to *SL* bloodstream infection in the true bacteremia group. None of the deaths in the contaminated group were attributed to *SL* infection. Of the 93 patients labelled true bacteremia, the source was most frequently listed as central line associated bloodstream infection (17.2%), followed by skin/soft tissue infection (11.8%), and infective endocarditis (IE) (10.8%).

Table 1. Characteristics of Patients with S. lugdunensis Positive Blood Cultures

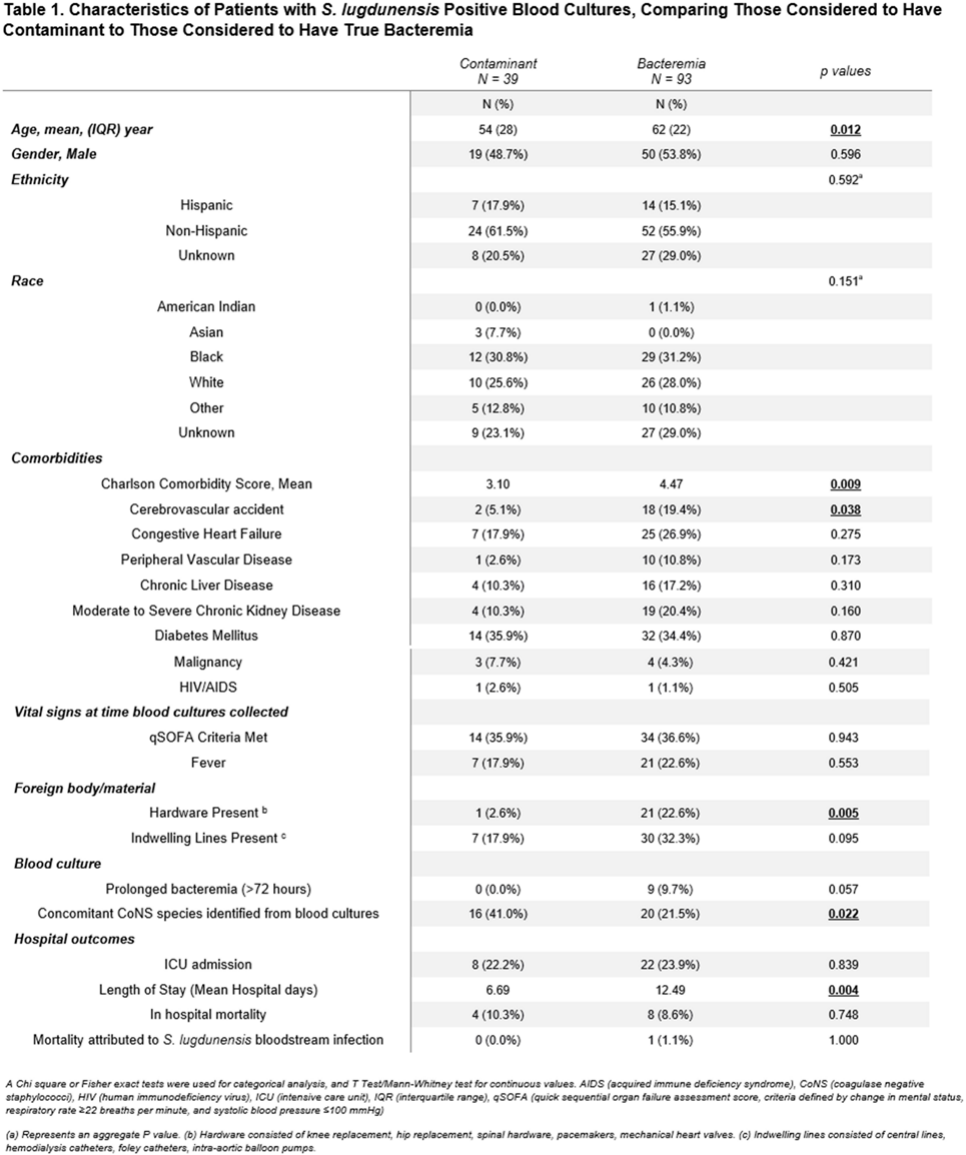

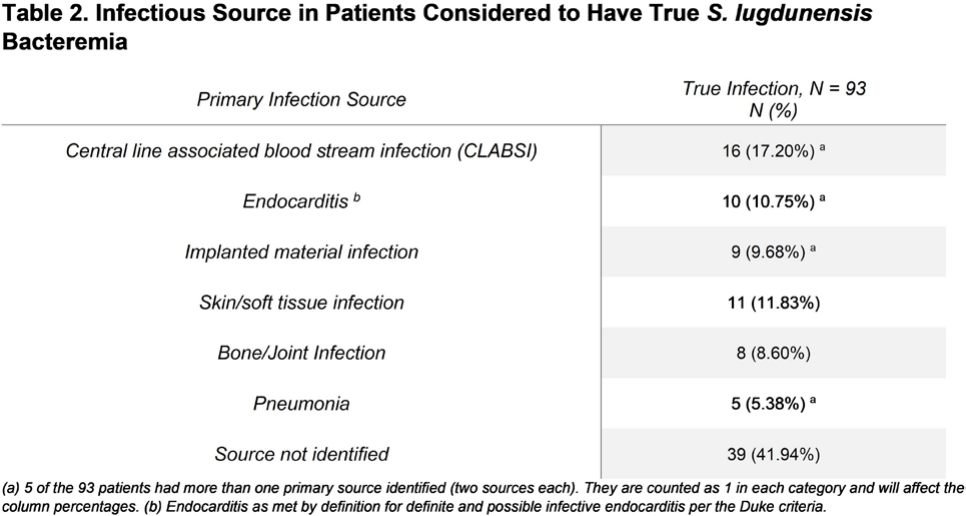

**Conclusion:**

In our study, 29% of patients with positive blood culture for *SL* were deemed contaminants. Patients without hardwares or positive concomitant other CoNS species from the same blood culture were often considered as contaminated cases. The incidence of IE remains as high as 10.8% in those patients identified to have true bacteremia (7.6% overall in our cohort), although lower than previously reported cases. Careful evaluation is warranted in patients with positive *SL* blood culture to rule out severe infections and avoid unnecessary courses of antibiotic therapy. This study suggests that increased identification of *SL* may impact our understanding of its significance and pathogenicity over time.

**Disclosures:**

**All Authors**: No reported disclosures

